# Health sector operational planning and budgeting processes in Kenya—“never the twain shall meet”

**DOI:** 10.1002/hpm.2286

**Published:** 2015-03-18

**Authors:** Benjamin Tsofa, Sassy Molyneux, Catherine Goodman

**Affiliations:** ^1^ KEMRI‐Wellcome Trust Research ProgrammeKilifiKenya; ^2^ Nuffield Department of MedicineUniversity of OxfordOxfordUK; ^3^ Global Health DepartmentLondon School of Hygiene and Tropical MedicineLondonUK

**Keywords:** operational planning, budgeting, priority setting

## Abstract

Operational planning is considered an important tool for translating government policies and strategic objectives into day‐to‐day management activities. However, developing countries suffer from persistent misalignment between policy, planning and budgeting. The Medium Term Expenditure Framework (MTEF) was introduced to address this misalignment. Kenya adopted the MTEF in the early 2000s, and in 2005, the Ministry of Health adopted the Annual Operational Plan process to adapt the MTEF to the health sector. This study assessed the degree to which the health sector Annual Operational Plan process in Kenya has achieved alignment between planning and budgeting at the national level, using document reviews, participant observation and key informant interviews. We found that the Kenyan health sector was far from achieving planning and budgeting alignment. Several factors contributed to this problem including weak Ministry of Health stewardship and institutionalized separation between planning and budgeting processes; a rapidly changing planning and budgeting environment; lack of reliable data to inform target setting and poor participation by key stakeholders in the process including a top‐down approach to target setting. We conclude that alignment is unlikely to be achieved without consideration of the specific institutional contexts and the power relationships between stakeholders. In particular, there is a need for institutional integration of the planning and budgeting processes into a common cycle and framework with common reporting lines and for improved data and local‐level input to inform appropriate and realistic target setting. © 2015 The Authors. *International Journal of Health Planning and Management* published by John Wiley & Sons, Ltd.

## Introduction

Planning helps to define a “journey” of where one wants to go and the road map and timeline for getting to the desired destination. Planning should always be undertaken with consideration of the amount of resources available, the competition for these resources, and the contextual factors within which resource allocation and prioritization occur. Thus, the description of the targeted destination in the journey ought to be a balance between the desire to get there and the reality of the resources available and the context within which these resources are allocated and managed (Bryson, 1988; Denis *et al.,* 1995; Green *et al.,* 2002).

Public sector planning is an important tool for translating government intentions and policies into activities on the ground. Planning in the public sector takes two broad forms: First, as a continuation of the policy making process, through long‐term sector strategic visioning and second, as a day‐to‐day management tool for operationalization of policies through short‐term operational plans. Public sector planning and budgeting not only should aim to ensure rationalization and prioritization in the use of limited available resources but also inevitably needs to respond to internal and external environmental factors, such as donor requirements, political interests, planning and budgeting institutional arrangements and society's social values (Mburu, 1994; Green and Mirzoev, 2008). In addition to these influences, health sector planning and budgeting specifically is imbued with the vested interests of different stakeholders, groups and individuals. These actors' roles should be viewed as part of the broader social, economic, political and general ideological context within which they operate (Mburu, 1994; Zaidi, 1994).

Interests and influences present a constant challenge in the alignment of public sector planning and budgeting, and within health sectors specifically, in many developing countries (The World Bank, 1998; Oxford Policy Management, 2000; Le Houerou and Taliercio, 2002; Allison, 2008; Muchiri, 2010). In an attempt to help countries address this challenge, in the early 1990s, the World Bank began to promote the Medium Term Expenditure Framework (MTEF) as a planning and budgeting tool designed to link public sector priority policy objectives and activities identified during planning with budget vote heads, in a concept referred to as “output‐based budgeting” (Oxford Policy Management, 2000). Since then, MTEF has been widely adopted by many developing countries, particularly in Sub‐Saharan Africa. Emerging evidence indicates however that its adoption and implementation have not necessarily led to better alignment between government policies, plans and budgets (Le Houerou and Taliercio, 2002). It has been argued that this has been due to countries using the MTEF as a standardized prescriptive budgeting tool, without an attempt to adapt it to local country level contextual factors influencing the planning and budgeting processes (Oxford Policy Management, 2000; Le Houerou and Taliercio, 2002). In the context of on‐going efforts to improve health sector planning and budgeting tools and outcomes, there is a need for more empirical understanding of the range of contextual issues affecting planning and budgeting processes in public sectors.

A lack of linkages between budgetary allocations and sector priorities have been cited as one of the reasons why Kenya has failed to achieve its health‐related targets (Glenngard and Maina, 2007; Ministry of Public Health and Sanitation and M. o. M. Services, 2009). As with many other developing countries, the government of Kenya (GoK) has for many years been undergoing major health systems reforms aimed at improving resource priority setting, planning and budgeting, including the involvement of communities and sub‐national level units in planning and budgeting decision‐making (Ministry of Health, 1994; Ministry of Health, 1999; Ministry of Health, 2000; Ministry of Health, 2005). Until implementation of national devolution began in 2013, the health sector was coordinated through three levels; the Ministry of Health (MoH) headquarters at the national level, the Provincial Health Management Teams (PHMTs) at regional level and the District Health Management Teams at district level (Ministry of Health, 2005; Ministry of Health, 2007). Since 2005, health services have been organized around a minimum care package known as the Kenyan Essential Package for Health (KEPH) (Ministry of Health, 2007), which outlines six services delivery levels—community unit, dispensary, health center, district hospital, provincial/regional hospital and national hospital—with service interventions at each level delivered as care packages targeting six population groups (Ministry of Health, 2007).

The three coordination and six service delivery levels have been the main sector planning units expected to undertake annual operational planning and budgeting in a bottom‐up fashion (Ministry of Health, 2005; O'Meara *et al.,* 2011). In 2008, following the disputed 2007 election results, Kenya formed a coalition government (Goverment of Kenya, 2008). The coalition government complicated the coordination of the health sector by splitting the MoH into the Ministry of Medical Services (MoMS) responsible for curative and rehabilitative health services and the Ministry of Public Health and Sanitation (MoPHS) responsible for preventive health services and health promotion (Goverment of Kenya, 2008).

The first “Kenya Health Policy Framework 1994–2010” was developed to guide health sector planning across levels and units (Ministry of Health, 1994). To facilitate its implementation, the MoH developed a 5‐year national health strategic plan in 1999 and a subsequent one in 2005. Although developed with clear priority objectives and targets, the implementation period of these strategic plans has been characterized by lack of linkage between the strategic priority objectives identified in the sector strategic plan with the annual itemized government budgetary allocations within the MoH. In the 2000/2001 fiscal year, the GoK adopted the MTEF as a tool for aligning public sector planning and budgeting (Philippe Le Houerou and Taliercio R 2002, Muchiri, 2010); and in 2005, the MoH adopted Annual Operational Plans (AOPs) as a way of adapting the MTEF process in the implementation of the national strategic plan for health (Ministry of Health, 2005; Muchiri, 2010).

We examined the AOP planning and budgeting processes at the national level in Kenya and the factors that influenced this process at the time. Data were collected in 2012, in the lead up to national elections. Our findings are relevant to ongoing efforts to improve health sector planning and budgeting in Kenya. They also contribute to the broader body of literature that seeks to understand public sector—including health sector specific—planning and budgeting processes and their influences in Sub‐Saharan Africa.

## Study Methods

This was a qualitative study with data collected by BT in Nairobi between February and September 2012, primarily through participant observation, document review and formal in‐depth interviews.

### Participant observation

Benjamin Tsofa was formally attached to the MoH headquarters in Nairobi for the 8‐month data collection period. Over this period, he attended all meetings and activities relating to the AOP planning and budgeting processes, observing formal and informal interactions by the key actors involved in the planning process at the MoH. As a former district manager in‐charge of health, he was assigned active technical roles within the MoH Technical Planning department, providing technical assistance and input in designing the AOP planning tools and guidelines. This provided him with an insider perspective, with access to information and operations of the MoH that would not be accessible to a purely external researcher (Dwyer and Buckle, 2009). To strengthen objectivity in the interpretation of his observations, regular formal reflective sessions were carried out with the other research team members to allow for group critique of the data and interpretations. Field notes were kept in the form of a diary throughout this period. The observation field notes, and deliberations from the reflection sessions, were triangulated with data from the key informant interviews and document reviews to minimize bias related to BT's insider status.

### Document review

All documents relating to the AOP and general planning and budgeting processes in the MoH were identified and their content reviewed to provide an understanding of the goals, intentions and intended process of the planning and budgeting cycle. These documents included the Kenya Health Policy Framework 1994–2010, the MoH Report on Health Sector Decentralisation 2000, the first and second National Health Sector Strategic Plans, the second National Health Sector Strategic Plan mid‐term, draft end‐term review reports, the MoMS Strategic Plan 2008–2012, the MoPHS Strategic Plan 2008–2012, the draft Kenya Health Policy 2012–2030 and the Ministry of Finance MTEF Manual 2011.

### Semi‐structured interviews

Following the observations and document review, 12 key informant interviews were conducted in English, with purposefully selected actors involved in the 2012–2013 fiscal year health sector planning and budgeting processes. Participants selected represented a wide range of individuals drawn from both MoMS and MoPHS Technical and Economic Policy and Planning departments and representatives from health sector Non‐Governmental Organizations (NGOs) at the national level under the umbrella of Health NGOs Network, Kenya; UN agencies such as World Health Organization (WHO), United Nations Children's Fund and World Bank; and bilateral health sector donor agencies including the Danish Agency for International Development and the United States Agency for International Development. Interviews aimed to explore how the key actors perceived and interpreted the health sector AOP planning and budgeting processes and the factors that influence these processes. All interviews were digitally recorded and transcribed verbatim. No invited participants refused to be interviewed.

We used the framework analysis approach described by Pope *et al*. (Pope *et al.,* 2007) for analysis, while incorporating concepts from the policy analysis triangle (Walt and Gilson, 1994) to facilitate a targeted exploration of the process, content, actors and context of the planning and budgeting processes.

## Findings

### The national health sector AOP and budgeting process on paper

In this section, we outline how the AOP process is supposed to be conducted on the paper, as described in policy documents and as understood by key actors involved in coordinating the process.

The MoH adopted the AOP in order to harmonize and integrate planning and budgeting processes in the health sector. The process should be conducted in a participatory manner by all stakeholders and under the leadership of the ministry. This intention is articulated in the second National Health Sector Strategic Plan

…this strategic plan will initiate a process of joint annual planning and budgeting under the leadership of the MoH, together with main stakeholders in the sector…—NHSSP 2—pg 47

**Figure 1:**
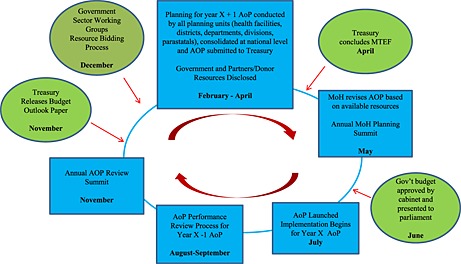
The ministry of health AOP planning and MTEF budgeting cycle on paper: the blue boxes represent AOP activities, while green circles represent MTEF budgeting activities

The year‐by‐year AOP priority targets are supposed to be guided by the sector strategic objectives, the preceding year's sector performance and available resources for the specific year. According to the policy documents, the AOP process and cycle should begin in November each year, at the AOP review summit where the sector identifies the priorities for the coming year (Figure 1). This summit is planned to coincide with the treasury releasing the Budget Outlook Paper that elaborates the respective sector budgetary ceilings.

**Figure 2:**
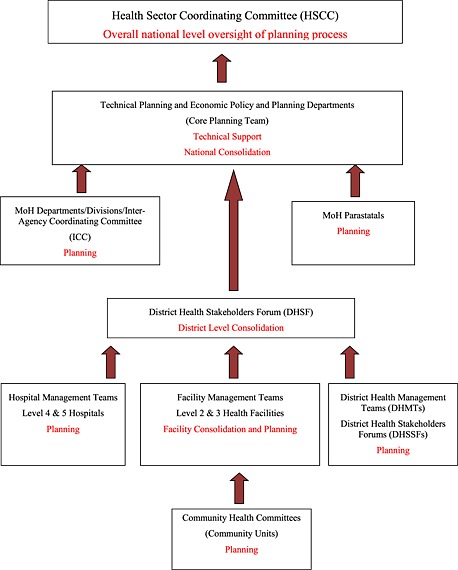
The main AOP planning activities and stakeholders involved

The MoH should use identified priorities from the AOP review summit to bid for resources at the hearings in the Sector Working Group then prepare AOP planning tools, guidelines and resource envelopes for planning units, based on the indicative government resources allocated from the Sector Working Group hearings and declared resources from donor partners. Planning units should begin planning in February in a “bottom‐up” fashion, from the lowest planning unit upwards (Figure 2). The MoH then submits the consolidated ministry AOP through the Sector Working Group in April for funding consideration by Treasury. Treasury finalizes the national budget process and communicates back to ministries the resources they have been allocated. The MoH should then revise its AOP based on resources confirmed by Treasury. In May, the MoH should organize the annual planning summit where stakeholders meet to discuss the work plan, which is later launched in June to begin implementation in July, once Treasury presents the national budget to parliament. The review of the previous year's AOP should begin in August. This should begin with all planning units preparing a short annual performance report following a template provided by ministry headquarters. These reports should be consolidated in a bottom‐up manner, similar to the planning process. The final MoH consolidated report forms the national AOP performance report, to be presented to partners and stakeholders by September. This report forms the agenda for stakeholders' discussions at the November AOP review summit.

National stewardship of the planning process should be provided by the Health Sector Coordinating Committee (HSCC; Figure 2), which comprises the MoH Heads of Departments, and representatives from key partners in health at the national level including UN agencies, for example, WHO, United Nations Children's Fund and United Nations Population Fund (UNFPA); donor government agencies, for example, United States Agency for International Development, Danish Agency for International Development and Japan International Cooperation Agency (JICA); and health NGOs. The Economic Policy and Planning department of the MoH is charged with outlining the resource envelope for planning units, while a Technical Planning department is to provide technical guidance for all planning units. The two planning departments jointly form the secretariat of the HSCC. Non‐government actors are supposed to participate in the priority setting, planning, financing and monitoring of the AOPs together with the MoH, through involvement in the District Health Stakeholder Forums, Inter‐Agency Coordinating Committees and the HSCC. The sector adopted a sector‐wide approach (SWAp) for joint planning, financing, implementing and monitoring of the AOPs. To emphasize the commitment to the SWAp principles by partners, a code of regulation and conduct was developed and signed by all key actors in the sector 2007.

### Implementation and key influences on the 2012–2013 AOP and MTEF processes in practice

In this section, we describe what actually happened during the 2012–2013 fiscal year planning and budgeting cycle and key influences on the observed processes.

#### Overview of the process, timelines and activities

Table 1 details the key activities and timelines for the 2012–2013 AOP and budgeting process, comparing them with what should have happened as described in the policy documents. In general, this indicates that several years after the adoption of AOPs in the health sector, the overall strategic goal of the AOPs for creating linkage between planning and budgeting is far from being realized. Most respondents interviewed also agreed that this mismatch between AOP and budgeting processes has been a problem and continues to be the biggest challenge to the AOP process in the sector.

…The main weakness of the AOP has been these two systems or these two planning processes have run independently of each other and they continue to do so today.—KII 003

**Table 1:**
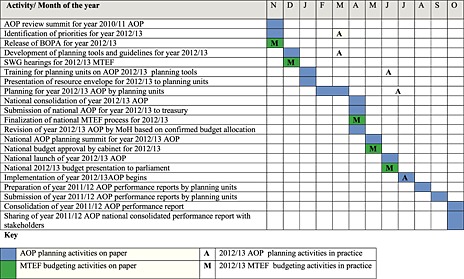
Summary of key AOP and MTEF activities illustrating when they should have happened and when they actually happened during the planning for 2012–2013 fiscal year

AOP, annual operational plan; MTEF, medium term expenditure framework; BOPA, Budget Outlook Paper; SWG, Sector Working Group.

In 2012–2013, the government‐wide MTEF process went on as scheduled. Treasury released the Budget Outlook Paper in November 2011, sector hearings were conducted in December, and the budget was concluded in April 2012, presented to cabinet for approval in May and finally presented to parliament on 14 June 2012, in line with the requirements of the country's constitution.

The AOP process was however significantly delayed. The review of the 2010–2011 AOP, which was to be done in November 2011 so as to inform the 2012–2013 priority setting and guide the MoH's resource bidding at the Sector Working Group, was never done. Instead, the 2012–2013 AOP development process started with production of draft planning templates and guidelines in December 2011. The template development was then shelved until March 2012 when it was finalized, as opposed to finalization in December as per schedule. This was then followed by incorporation of the planning tools into the District Integrated Health Information System (DHIS) in April to allow for online submission of plans by planning units for the first time. The tools were then field piloted in one district in May 2012, also for the first time, and adopted thereafter.

All PHMTs and representatives from all departments and divisions in the two ministries were taken through a 2‐day training and orientation on the new planning tools on the 27th and 28th of June 2012. The PHMTs were then tasked to train the district teams. The templates included a predetermined list of indicators on which planning units would be required to report and their performance would be measured. Based on the predetermined indicators, the Technical Planning department drew up targets and distributed them across all the provinces. The PHMTs were then tasked to distribute the targets to their respective districts using the same criteria. All planning units were required to begin planning and to submit their plans by the end of July 2012, by uploading them onto the DHIS, but this happened very slowly. The end result was that by the beginning of July, when AOP implementation is supposed to begin, the health sector had a budget but no AOP.

Although the AOP process is intended and described as a “bottom‐up” process, in practice, the process was heavily “top‐down” driven. In general, all the planning templates were developed at the national level with no input from sub‐national peripheral levels. There was notably no link made between resource availability and the targets set, and there was no participation of non‐government actors in the target setting.

We now describe four key influences on the planning and budgeting processes, which led to their persistent misalignment: stewardship and coordination challenges, the rapidly changing organization and planning environment, data use for target setting during planning and low uptake and participation by planning units.

#### Stewardship and coordination

A number of key stewardship and coordination factors influenced the planning and budgeting processes. First, the functioning of the HSCC was sub‐optimal. During the entire 2012–2013 AOP planning period, attendance of HSCC meetings by many Department Heads was poor, and there was lack of follow‐up on issues discussed and agreed at the meetings. This was expressed as a concern by most participants interviewed, as illustrated here

…and really I think the delink between what is in theory and what is actually practiced I think in my view it's a reflection of the stewardship gaps that exist in the sector at the moment—KII 001

To improve coordination of the planning and budgeting processes, a “Core Planning Team” was created by the Technical Planning and Economic Planning departments of the two ministries, together with WHO technical advisors and selected technical officers from a few non‐government partners. The Core Team worked to coordinate the planning process. However, this did not solve all the problems with the operation of the HSCC. The membership of the Core Team was not explicit, and thus, people could be invited in and drop out at different times. The team also lacked explicitly defined terms of reference and had no clear leadership structure or clear reporting responsibilities to the HSCC.

A second key stewardship issue was the institutionalized separation between planning and budgeting processes. The government‐wide MTEF process is a legally entrenched process with specific timelines. Treasury is thus legally accountable to the cabinet, parliament and the citizenry and has to ensure that the government budget is drawn every year irrespective of whether specific sectors participate actively or not. Within the MoH, the Economic Policy and Planning department that coordinates the MTEF budgeting is headed by a Chief Economist who is seconded from and thus accountable to Treasury. Meanwhile, the Technical Planning department that coordinates the technical AOP is accountable to the Technical Director in the MoH. This has continuously raised bureaucratic challenges and hampered planning and budgeting harmonization efforts. There was consensus among most of the respondents interviewed that these two teams need to be merged or at least work under one accountability structure if harmonization of planning and budgeting is to be realized.

….Ideally, this is my own thinking…all the planning should be under one department. It should be one unit, actually…and still you can tape this; I know there has been a fight; people saying you have taken the AOP from us yeah,…Silent, silent fight yeah always…—AOP KII 004

For example, the tools training and orientation meeting for PHMTs was largely organized by the Technical Planning department, and despite being asked to, the Economic Policy and Planning department did not come to share the government health sector resource envelope with the planning teams at the training. The Technical Planning team could not hold them accountable for this. This was despite the budgeting process having been concluded in April and the national budget having already been presented to parliament by Treasury. Thus, the Economic Policy and Planning department was aware of the government's actual allocations to health.

A final stewardship issue was related to the role of non‐government partners in the planning process. Although all donor partners and other non‐state actors in the sector committed themselves to the health SWAp by signing the code of regulation and conduct, most of these actors were minimally involved in the 2012–2013 AOP process. Apart from the few technical officers co‐opted into the Core Team from three organizations, and the WHO country office, the only other involvement of partners was when the Core Team required funding to facilitate some activities in the process. Quite striking was the observation that the MoH did not have a set budget/resource allocation to facilitate the planning process and therefore had to rely entirely on non‐government partners. This observation was echoed by some interviewees

…Then the issue of finances, in my time as a part of the coordinating team I have not seen the government; and the government here is the ministries of health, putting money for planning process…—KII 004

#### Rapidly changing organizational and planning environment in the health sector

The existence of the two ministries after the split of the former MoH in 2008 into MoMS and MoPHS caused major challenges by complicating the stewardship and coordinating roles of the AOP process by the MoH. Several participants echoed the concern that the existence of the two ministries of health had complicated the coordination role of the central MoH and had thus compromised the planning process

…Now, the two ministries really after the split, coordination of the planning process became a little bit challenging because of the bureaucracy…—KII 012

*…I think* (the relationship between the two ministries has been) *chaotic,…sorry to use that word…It has really compromised the whole planning processes. okay, so we don't see ourselves as a sector any more, we see ourselves as the vertical ministries…—KII 002*

At the time of undertaking, the 2012–2013 AOP process, the MoH was also developing a new national health policy, the Kenya Health Policy 2012–2030, and a new 5‐year strategic plan. Both the draft policy and strategic plan documents proposed the merger of the two ministries back into one MoH, in line with the 2010 constitution, which had placed a ceiling on the number of ministries the government could create after the general elections scheduled in March 2013. The proposed new MoH was to be lean, as all operational functions were to be devolved to the counties. From observations, there was a high degree of uncertainty in both ministries as many senior officers, including those who were managing and coordinating the planning process, were unsure what would happen to their positions after the proposed MoH merger and the devolution.

Another direct consequence of the changing planning and organizational environment on the 2012–2013 AOP was the delay in finalization of planning templates and tools, from December 2011 to March 2012. This was because the MoH was still not clear how the health services would be organized, as both the new national health policy and strategic plan were being developed. For example, in 2012–2013, important changes were required to the planning templates to reflect revisions to the Kenya Essential Package of Health (KEPH) as part of the development of the Kenya Health Policy 2012–2030. This involved revising the levels of care to fit with the newly devolved structure and introducing a set of specific policy objectives. The development of the AOP planning templates was resumed after the new KEPH had been agreed on within the new health policy and the new strategic plan development framework.

#### Data use in priority and target setting

During the planning process, the specific priorities and targets for each AOP are to be derived from the national health strategic plan and determined by the preceding year's AOP review. However, some participants interviewed felt that the quality of routinely collected data was questionable and could not be relied on for monitoring the AOP implementation and for informing target setting during the planning process. More so, the 2012–2013 AOP had a major change in strategic focus from the previous years, as it was based on the new KEPH. Specific indicators were designed around these new strategic focus areas, and targets were set by the Technical Planning department. There were very poor, or in most cases no baseline data around most of the indicators selected. This led to a feeling by most participants interviewed, that the planning and target setting lacked an objective basis

…it lacks objectivity in the sense that a lot of the things that ends up as priorities for various levels of the health sector and also in various planning units if you may, really depends a lot on boardroom gut feeling…—KII 008

*…Ooh* (laughs) *I don't think anybody knows exactly how it's done* (laughter) *somehow targets appear but just…okay what I would describe* (is the) *ideal situation in actual practice it's total, it's total chaos. One* (issue) *is that we have a problem in that we do not have good data to tell us exactly what is being achieved at present, so when it comes to setting targets then it becomes very difficult…—KII 001*

#### Uptake and participation by planning units

After the training and orientation, the PHMTs and ministry departmental teams were asked to ensure that all planning units finalize and upload their plans onto the DHIS by the end of July 2012. However, by the end of the first quarter of AOP implementation at the end of September 2012, the submission of AOPs by planning units onto the DHIS was very low, with plans uploaded by only 1600 of the 2025 community units, 1118 of the 6526 dispensaries, 331 of the 1164 health centers, 197 of the 493 county hospitals and 19 out of the 265 District Health Management Teams. At the national level, only three of the 17 technical departments of the two ministries and six of the 32 divisions and programs had uploaded plans. None of the four national hospitals and the six health sector parastatals had uploaded their plans.

This partly reflected a shift in focus of the Core Team and the HSCC after July 2012 to the development of the new strategic plan, with no subsequent visits by any Core Team members to the peripheral sites to offer technical support and facilitate the planning process as has been done in previous years during AOP development.

Moreover, although the AOP process was intended and described as a “bottom‐up”, in practice, the process was heavily “top‐down” driven. In general, all planning templates were developed at the national level with no input from sub‐national peripheral levels. Targets for provinces were set at national level based on their population sizes, without consulting with the provinces as to whether they would be in a position to attain them. The PHMTs were then tasked to distribute the targets to their respective districts using the same criteria. There was notably no link made between resource availability and the targets set, and there was no participation of non‐government actors in the target setting. This is likely to have undermined the legitimacy of the targets and the overall planning process at a local level.

## Discussion

When the World Bank introduced and advocated for the adoption of the MTEF in the early 1990s, it was viewed as the “magic bullet” that would solve the problems of misalignment between planning and budgeting (Philippe Le Houerou and Taliercio R 2002). For the same reasons, the GoK adopted the MTEF, and subsequently the AOP process, as a way of linking technical planning with budgeting within the MoH. However, from the findings of this study, the desired linkage between policy, planning and budgeting is far from being realized several years since the adoption of these structured planning and budgeting processes. Several factors including sector stewardship and coordination, the rapidly changing institutional and planning environment, non‐reliable available data to inform priority setting and poor stakeholder participation have all contributed to the challenges of implementing the AOP planning and budgeting policy in the health sector, as designed in the policy and strategic documents.

The findings of this study are not unique. In their study to compare MTEF implementation experience in 13 African countries, Le Houerou and Taliericio found that MTEF implementation has had a minimal impact in achieving public sector planning and budgeting harmonization in these countries (Philippe Le Houerou and Taliercio R 2002). They observed that this lack of successful implementation has been due to more attention being paid to the technical aspects of the MTEF, which assumes that there is always a rational linear linkage between policy objectives, priority activities and budget line items during planning and budgeting in public sectors. Le Houerou and Taliericio argued that paying attention to political and institutional management systems that are in place to oversee the planning and budgeting processes within country, and within different sectors in the same country, is a key for successful harmonization of public sector planning and budgeting.

Our findings agree with Le Houerou and Taliericio's observations. Within the MoH in Kenya, Treasury seconded economists accountable to Treasury to oversee the MTEF process, with minimal consideration on how they would work and integrate with the technical team in‐charge of planning in the MoH. The lack of integration and common accountability channels between these economists from Treasury, and the technical planning team coordinating technical planning in the MoH, has largely seen the MTEF conducted and perceived within the MoH as an externally driven process. This has meant that the linkage with the technical planning process has remained elusive. From our findings, most key actors in the health sector believe that integration of the Technical Planning and the Economic Policy and Planning unit should be the first step taken if efforts to align planning and budgeting are to be successful. This perception is consistent with observations by Allison (Allison, 2008), who found that the establishment of integrated health sector planning and budgeting teams in some selected states in Nigeria, with the support of the Partnership for Transforming Health Systems project, led to successful integration of planning and budgeting within those states.

Notably, both the national political environment and the internal MoH environment were very different around the time of the adoption of the AOP policy (2004/2005), from the 2012–2013 AOP planning period. The AOP policy was designed and adopted in the early days of a popularly elected National Rainbow Coalition (NARC) government that had replaced many years of the previous unpopular leadership. The newly elected NARC government, while enjoying high levels of public goodwill, was keen to correct the perceived poor policies of the former regime (Kagwanja and Southall, 2009). Within the health sector, NARC had one MoH, and the minister in‐charge was one of the key leaders of the coalition that had formed the government. In contrast, in 2012, the sector had two Ministries of Health, each belonging to one side of a coalition government formed in 2008 that had been characterized by internal wrangles and uncoordinated running of government affairs since its formation (Kagwanja and Southall, 2009; Ogosia *et al.,* 2009, February 27th, Cheeseman and Tendi, 2010). These two contrasting broader political contexts could partly explain how the rapidly changing organization and political environment has negatively affected the achievement of the desire for planning and budgeting harmonization in the MoH.

The 2012–2013 AOP planning process was also atypical in several ways. The AOP was being developed when the national health strategic plan and national health policy period had expired. There was thus no national health policy and strategic plan to provide a clearly defined sector strategic direction to be implemented by the AOP. The country and the health sector were also going through the initial stages of implementing the new constitution, particularly the planned devolved government structures, and yet, the necessary subsidiary laws for guiding the process had not been fully enacted, creating a sense of uncertainty both in the MoH and the country at large. Although adaptation of new policies and political confusion and uncertainties is not unusual in health systems, these features will have been relatively heightened at this particular time of transition in Kenya, and hence, the findings of this study need to be interpreted in that light.

Walt and Gilson have argued that actors both as individuals and as institutions are a central core in the policy environment, hence, understanding their roles is a key to understanding policy dynamics (Walt and Gilson, 1994). The AOP policy guidelines identified the key actors and institutions and their respective roles at the different stages of the planning cycle. The policy intent was to make the process very consultative, participatory and transparent at all levels, and thus, it adopted the “bottom‐up” and SWAp approaches to ensure inclusivity for all stakeholders. A major assumption of the policy guidelines was that MoH headquarter actors are a single homogeneous group that acts as a unit to oversee and provide stewardship for process. In practice, however, the MoH headquarters are made of different groups of “policy elites” (Buse *et al.,* 2005) who at different stages exert their different forms of power to facilitate or impede the process through their deliberate actions or in‐actions. This was particularly evident from the findings of this study where the Economic Policy and Planning team went ahead with the MTEF budgeting process without any consideration of the Ministry's technical plan and without participating in the AOP development process.

Both MoMS and MoPHS appeared on the paper to have a very hierarchical organogram with definite reporting lines (Ministry of Medical Services, 2008; Ministry of Public Health and Sanitation, 2008). However, in practice, individuals within the structure wield significant individual technical power, giving them a very high degree of independence from, and minimal accountability to, the hierarchy. This was typically illustrated by the lack of participation by most Department Heads in the HSCC meetings and other planning activities and minimal ability by the system to hold them accountable. The operations of the Core Team were also rather amorphous with no clear responsibility and accountability mechanism. These observations partly explain why—despite both the Technical Planning and Economic Planning teams being members of the Core Team—coordination and communication were challenging, with negative implications for the successful implementation of the MTEF and AOP in 2012–2013.

Both the MTEF and AOP are described on the paper as bottom‐up processes, with the aim of making government planning and budgeting participatory and transparent. In practice, however, the 2012–2013 AOP process was heavily “top‐down”. This finding suggests the reluctance of central level policy makers to undertake genuine decentralization of decision‐making within the planning process despite this being a core objective of the health sector reforms in Kenya (Oyaya and Rifikin, 2003; Wamai, 2009). The observation is consistent with that made by O'Meara and colleagues, who found the AOP process in Kilifi district in Kenya to be heavily influenced by top‐down push through the use of very prescriptive planning tools with predetermined national indicators. This push made it difficult for peripheral facilities to plan for identified local priorities even after engaging communities (O'Meara *et al.,* 2011).

To some degree, one may expect alignment between policy, planning and budgeting to improve over time as the major political changes at a national and sectoral level become established, reflecting the fact that the year 2012–2013 was a particularly turbulent one for policy makers. However, the aforementioned analysis indicates that many of the causes of non‐alignment are more structural and embedded in the Kenyan system, given both the nature of the institutions involved and the power dynamics between them. There is a clear need for integration of the MTEF budgeting process into the internal ministry specific planning systems so as to enhance alignment between planning and budgeting in public sectors. In particular, this is likely to require institutional integration of the teams undertaking technical planning and those undertaking budgeting or economic planning under one unit, with a common cycle and framework and common reporting lines. High level support and commitment for these structures and their systematic functioning will be required between both senior MoH and Treasury officials and between key donors and partners including the WHO and WB country officials. In addition, steps are required to ensure that accurate data are available to inform target setting, together with input from local planning units to inform the feasibility of target achievement. These steps will be essential if realities in the health sector are to inform policy and planning and if planning is to inform sector budget formation, rather than vice versa. Some progress has already been made in this regard. For example, new planning tools being developed by the MoH have deliberately been merged into one overall tool, which has been designed to reflect a continuum from performance review of the previous year's AoP, through technical priority elaboration and planning for the coming year's AoP and budgeting for the AoP. County Health Management Teams are currently being introduced to these revised planning tools and their rationale. The implementation and impact of these revisions need tracking over time and across counties, which are the focus of planning under devolution.

## Conclusions

This study contributes to the empirical literature on the challenges facing efforts to align policies, plans and budgets in public sectors including health. As in many countries, the Kenyan health sector is far from achieving planning and budgeting alignment, several years after the adoption of MTEF and AOP tools to address this. Several factors contributed to this problem including weak stewardship by senior MoH officials, institutionalized separation between planning and budgeting processes, a rapidly changing planning and budgeting environment, lack of reliable data to inform target setting and poor participation by key stakeholders in the process including a top‐down approach to target setting. In agreement with the existing literature, we conclude that the attainment of good intentions set out in the MTEF policy within the health sector cannot be achieved through the application of the MTEF process in a prescriptive manner without considering both the broader and health sector specific institutional context and the power relationships between stakeholders. In particular, there is a need for stronger commitment and stewardship for the planning and budgeting processes by senior MoH officials, institutional integration of the planning and budgeting processes into a common cycle and framework with common reporting lines and for improved data and local‐level input to inform appropriate and realistic target setting.
